# Beyond Methane Oxidation: The Protein Landscape of ANME‐2a Reveals an Integrated System for Diazotrophy and Membrane Fortification

**DOI:** 10.1111/1758-2229.70233

**Published:** 2025-11-17

**Authors:** Samuel de Souza e Silva, Natanael Borges de Avila, Alisson William da Silva, Lucas Ramos Fernandes da Silva, Matheus Ribeiro Prado, Murilo Alves Beppler, Anderson Rodrigues dos Santos

**Affiliations:** ^1^ Biotechnology Institute, Federal University of Uberlândia Uberlândia Brazil; ^2^ Faculty of Computing Federal University of Uberlândia Uberlândia Brazil; ^3^ Information Management Department Federal University of Uberlândia Uberlândia Brazil

**Keywords:** anaerobic methane oxidation (AOM), ANME‐2a, archaeal lipids, diazotrophy, metabolic modularity, niche adaptation, protein network

## Abstract

While renowned for mitigating methane emissions via anaerobic oxidation of methane (AOM), the full ecological strategy of ANME‐2a archaea still requires further exploration. This study looks beyond methane oxidation to map the protein landscape of ANME‐2a, revealing an integrated system for metabolic autonomy and environmental resilience in this specific isolate. Using a feature‐based protein network derived from 230 Methanosarcinales genomes, we uncovered a sophisticated modular architecture. Key findings demonstrate that the AOM machinery is not isolated but functionally coupled with distinct modules dedicated to auxiliary functions. Our analysis not only confirms that this ANME‐2a isolate possesses the complete genomic toolkit for autonomous diazotrophy but also reveals the molecular blueprint for its integration with AOM. We show how key machinery has been specialised to support the organism's core energy metabolism, with its core nitrogenase components co‐clustered within the same functional module as AOM electron transfer proteins. Furthermore, we identified a specialised module dedicated to ‘membrane fortification’ through the significant enrichment of pathways for archaeal lipid biosynthesis. This modular blueprint, which integrates core energy metabolism with nitrogen fixation and structural lipid production, provides a model for how diazotrophic ANME‐2a lineages may thrive as robust, self‐sufficient organisms adapted to dynamic, resource‐limited ecosystems.

## Graphical abstract

A protein interaction network of ANME‐2a uncovers a modular blueprint where core methane oxidation is energetically coupled to nitrogen fixation and membrane lipid biosynthesis. This integrated system explains the archaeon's metabolic autonomy and resilience in its extreme deep‐sea niche.

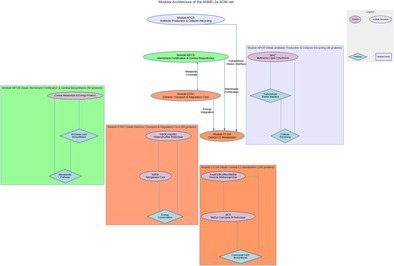

## 1 Introduction

The anaerobic oxidation of methane (AOM) is a vital biogeochemical process essential to the global carbon cycle, which helps regulate atmospheric methane emissions, a potent greenhouse gas (Reeburgh 2007). This process is initiated by the methyl‐coenzyme M reductase (MCR) complex, which catalyses the first and rate‐limiting step of methane activation (Thauer 2019). This critical enzyme reverses the final step of methanogenesis, converting methane and coenzyme M to methyl‐coenzyme M and coenzyme B, thereby initiating the entire AOM pathway (Hallam et al. 2004). Specialised microbial consortia carry out this process, primarily in anoxic marine environments, such as ocean sediments and deep lakes (Boetius et al. 2000; Knittel et al. 2005). AOM oxidises a significant amount of methane before it can reach the atmosphere, acting as a natural biological filter that mitigates atmospheric methane emissions (Hinrichs and Boetius 2002). Estimates suggest that AOM consumes approximately 70–300 Tg of methane per year, representing approximately 7%–30% of global methane production (Reeburgh 2007). Besides its climate impact, AOM also influences the biogeochemical cycles of other elements, such as sulfur and nitrogen, through its interactions with different electron acceptors, such as sulfate, nitrate or metal oxides (Knittel and Boetius 2009, Haroon et al. 2013, Cai et al. 2018). Understanding the molecular mechanisms and metabolic pathways involved in AOM is crucial for predicting and modeling future changes in methane fluxes in response to global climate change, as well as exploring potential biotechnological applications in mitigating atmospheric pollution emissions (Valentine 2002).

**FIGURE 1:**
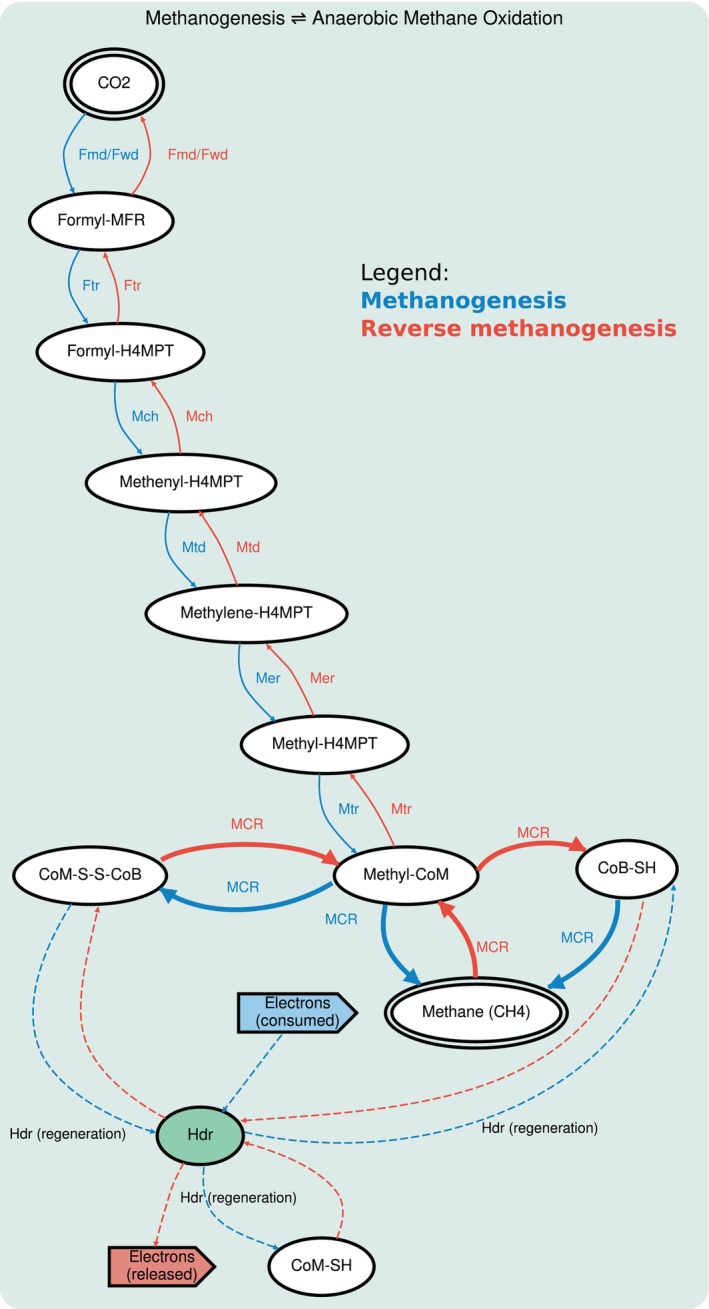
Schematic comparison of canonical methanogenesis and anaerobic methane oxidation (AOM). The diagram shows both pathways operating on the same enzymatic machinery but in opposite directions. Blue arrows represent the canonical methanogenesis pathway (CO_2_ → CH_4_), which consumes electrons to produce methane. Red arrows represent the AOM pathway (CH_4_ → CO_2_), which operates as the reverse of methanogenesis, oxidising methane and releasing electrons. Both processes utilise the central Methyl‐Coenzyme M Reductase (MCR) complex as the key catalytic step.

The biochemical foundation of AOM in ANME archaea is the remarkable process of reverse methanogenesis (Hallam et al. 2004). This pathway utilises the same core enzymatic machinery as canonical methanogenesis but operates in the reverse direction, consuming methane to produce CO_2_ and releasing electrons. This reverse operation is thermodynamically enabled by the unique conditions of methane seeps, such as high partial pressures of methane and the efficient consumption of electrons by diverse electron acceptors syntrophically (Knittel and Boetius 2009, Haroon et al. 2013, Cai et al. 2018). Besides its climate impact, AOM also influences the biogeochemical cycles of other elements, such as sulfur and nitrogen, through its interactions with these diverse electron acceptors by syntrophic partner bacteria (Thauer 2019). The key enzyme that catalyses the initial, rate‐limiting step of methane activation, the MCR complex, is central to both processes. Understanding how this core oxidative module is integrated with other cellular functions is therefore key to deciphering the ecological strategy of ANME‐2a (Figure 1).

At the forefront of the AOM process are the anaerobic methanotrophic archaea (ANME), which are diverse and widely distributed in methane‐rich environments like cold seeps (Knittel et al. 2005). The ANME‐2 subgroup has been identified as one of the most abundant ANME clades in marine sediments (Timmers et al. 2017). ANME‐2 archaea can reverse the methanogenesis pathway, effectively oxidising methane under anoxic conditions (Wegener et al. 2008), typically in syntrophic partnerships with sulfate‐reducing bacteria (SRBs) (McGlynn et al. 2018). However, ANME‐2 exhibits significant metabolic versatility, utilising alternative electron acceptors like nitrate (Haroon et al. 2013; Ettwig et al. 2010), iron (Cai et al. 2018, Beal et al. 2009) or manganese (Leu et al. 2020; Raghoebarsing et al. 2006; Beal et al. 2009; Metcalfe et al. 2021; Ettwig et al. 2010; Haroon et al. 2013) and possessing novel pathways for energy conservation and carbon assimilation (Beckmann et al. 2021, McGlynn et al. 2015), with autotrophy being a significant mode of carbon fixation in these communities (Kellermann et al. 2012). Furthermore, detecting active *nifH* gene expression suggested potential nitrogen fixation capabilities (Metcalfe et al. 2021, Dekas et al. 2009, Orphan et al. 2002), hinting at a broader ecological role. This is particularly relevant as methane seep ecosystems are often nitrogen‐limited, making in situ nitrogen fixation a critical capability for biomass production (Strous et al. 2006; Nawaz et al. 2025). However, how this energetically demanding process of nitrogen fixation is biochemically integrated with and energetically fueled by the core AOM pathway at the molecular level has remained a key unanswered question.

Despite progress in understanding the core reverse methanogenesis pathway (Hallam et al. 2004; Kruger et al. 2003) and associated electron transfer components (McGlynn et al. 2015, Meyerdierks et al. 2010), significant knowledge gaps remain regarding the full metabolic repertoire supporting AOM, the mechanisms enabling metabolic flexibility, the nature of ecological interactions beyond canonical syntrophy, and the regulation of these processes (Timmers et al. 2017; Leu et al. 2020, Hatzenpichler et al. 2016). Exploring the genomic potential for functions beyond core AOM is crucial for understanding the ecology and biogeochemical impact of ANME‐2.

While, to our knowledge, a dedicated protein–protein interaction (PPI) network has not previously been constructed for any ANME archaeon, computational methods for predicting PPI landscapes have become powerful tools in systems biology. Modern approaches leveraging Graph Neural Networks (GNNs) and integrating diverse data sources from databases like STRING have proven effective for mapping complex interactomes from sequence and structure information (Zhang et al. 2024). Concurrently, recent studies employing genomics and transcriptomics are beginning to illuminate the metabolic ‘parts list’ of these organisms. For example, studies on ANME lineages coupled to the reduction of iron (Slobodkin et al. 2023) and manganese (Leu et al. 2020) have consistently highlighted the central role of multiheme cytochromes (MHCs) in these processes. However, a systems‐level understanding of how these diverse metabolic capabilities are organised and functionally integrated remains elusive. A PPI network provides a powerful framework to map these functional connections and generate new hypotheses about metabolic modularity.

Our systems‐level bioinformatic approach combined PPI network inference, network topology analysis (modularity), pathway enrichment analysis, pathway completeness assessment based on key enzymes, and manual curation of interactions to investigate the extended metabolic capabilities and functional organisation associated with AOM in ANME‐2a (MAG: Candidatus Methanocomedens sp. isolate S7142MS1 2a_fsr_manual, whole genome shotgun sequence, accession number GCA_009649835.1). Using the GenPPi software (Anjos et al. 2021) on 230 Methanosarcinales genomes, we constructed a feature‐based PPI network for ANME‐2a. From this, we analysed a subnetwork (AOM net) centred on key core AOM proteins and their direct interactors. Clustering this AOM network revealed a modular structure involving these proteins, with canonical AOM components themselves being distributed among distinct modules—a key aspect we explore. Our subsequent analyses aimed to (1) elucidate the functional organisation of the AOM‐associated interactome, considering this inherent modularity of the AOM pathway, using statistical pathway enrichment and network analysis; (2) rigorously assess the genomic potential and completeness of key metabolic pathways, including nitrogen fixation, isoprenoid/lipid synthesis, and cofactor biosynthesis; (3) identify specific proteins and interactions potentially involved in auxiliary AOM support, bioenergetics, regulation and ecological interactions and (4) develop a revised hypothesis for ANME‐2a's metabolic versatility based on integrated evidence. Our findings reveal a robust potential for nitrogen fixation and highlight that not only auxiliary functions but also the AOM machinery itself is modularly organised. This organisation integrates core AOM processes with diverse adaptive capabilities, providing a more nuanced understanding of ANME‐2a's functional role in methane seep ecosystems.

## 2 Experimental Procedures

### 2.1 Genomes

The GenPPi software infers PPIs based on conserved genomic features across a set of related organisms. We selected the order Methanosarcinales as our reference group because ANME‐2 archaea are phylogenetically nested within this order, making it the most appropriate and closely related clade for comparative analysis (Timmers et al. 2017). We downloaded all available protein FASTA files from the NCBI database for species within this order, prefixed with the texts ‘ANME’ and ‘Meth’, comprising 230 species. The reference genome for ANME‐2a used for detailed analysis was *Candidatus Methanocomedens* sp. isolate S7142MS1 (GenBank assembly accession GCA_009649835.1). This Metagenome‐Assembled Genome (MAG), while fragmented into 247 scaffolds (Scaffold N50 of 9.1 kb), is supported by very deep sequencing coverage (300×), providing high confidence in the quality of the 1887 predicted protein‐coding genes used for our proteome‐wide analysis.

### 2.2 Interaction Network

We ran the GenPPi software (Anjos et al. 2021) over the 230 genomes represented by their protein FASTA files. The software predicts interactions based on evidence of functional association, derived from conserved gene neighbourhoods and co‐evolutionary patterns (phylogenetic profiles). While these functional links often imply direct physical binding, the method itself does not distinguish between the two. Consequently, the interactions presented in this study should be interpreted as strong functional associations—indicating that proteins are part of the same complex, pathway or process—rather than as confirmed physical binding events. Our strategy for minimising false positives relies on requiring strong genomic evidence for any predicted interaction.

This study deliberately utilised the ‘Features’ mode of GenPPi 1.5. This mode assesses protein similarity based on a direct comparison of 60 biophysical features derived from amino acid propensity indices, rather than the more sensitive Machine Learning (RF) model also available in the software. Our selection of this more conservative approach stemmed from two strategic considerations: first, generating a sparser, higher‐confidence network by focusing on stronger similarity signals; and second, ensuring the resulting network was computationally tractable for the detailed manual curation central to our analysis. A full description of the software, its different operational modes, and performance metrics is available in our dedicated methods paper (Silva et al. 2025). This strategy enabled analysis using 100 gigabytes of RAM and running 40 threads in under a week. The final feature‐based interaction network for ANME‐2a comprises 1869 proteins and 43,443 interactions (8767 neighbourhood‐based, 35,028 phylogenetic profile‐based). The network's node degree distribution followed a power law consistent with biological networks (Min: 1, Median: 16, Mean: 46, Max: 227). Details of the degree distribution analysis are provided in Supplementary Material A. It is important to note that this final network for our reference ANME‐2a isolate is a projection from the comprehensive analysis of all 230 Methanosarcinales genomes. The underlying evidence, such as phylogenetic profiles, is built from the entire genomic dataset, ensuring that the predicted interactions are those conserved across the broader phylogenetic order.

Additionally, we generated a second, denser PPI network using a machine learning‐based approach within GenPPi, distinct from the feature‐based method. This network encompasses 1887 protein nodes and 232,217 predicted interactions, exhibiting a higher average node degree (Min: 9, Median: 30, Mean: 246.1, Max: 633) than the feature‐based network. While we primarily used the sparser feature‐based network for global analyses, such as modularity, due to its reliance on stronger genomic and evolutionary evidence and better tractability, we consulted the machine learning‐based network as a complementary resource to explore specific hypotheses, such as potential interactions between nitrogen fixation components and the AOM machinery.

### 2.3 The AOM Subnetwork Construction

To study the processes directly linked to AOM, we derived a subnetwork (AOM net) from the whole‐genome feature‐based PPI network. We selected the 34 proteins (target proteins) encoded by 12 well‐studied genes/gene clusters central to AOM, with particular emphasis on the MCR complex as it catalyses the initial methane activation step that defines the AOM process (Fmd, Fpo/Fqo, Ftr, HdrABC, HdrDE, Mch, Mcr, Mer, MHC, Mtd, Mtr, Rnf). The AOM net included these 34 target proteins plus all of their immediate neighbours (proteins connected by at least one edge).

**FIGURE 2:**
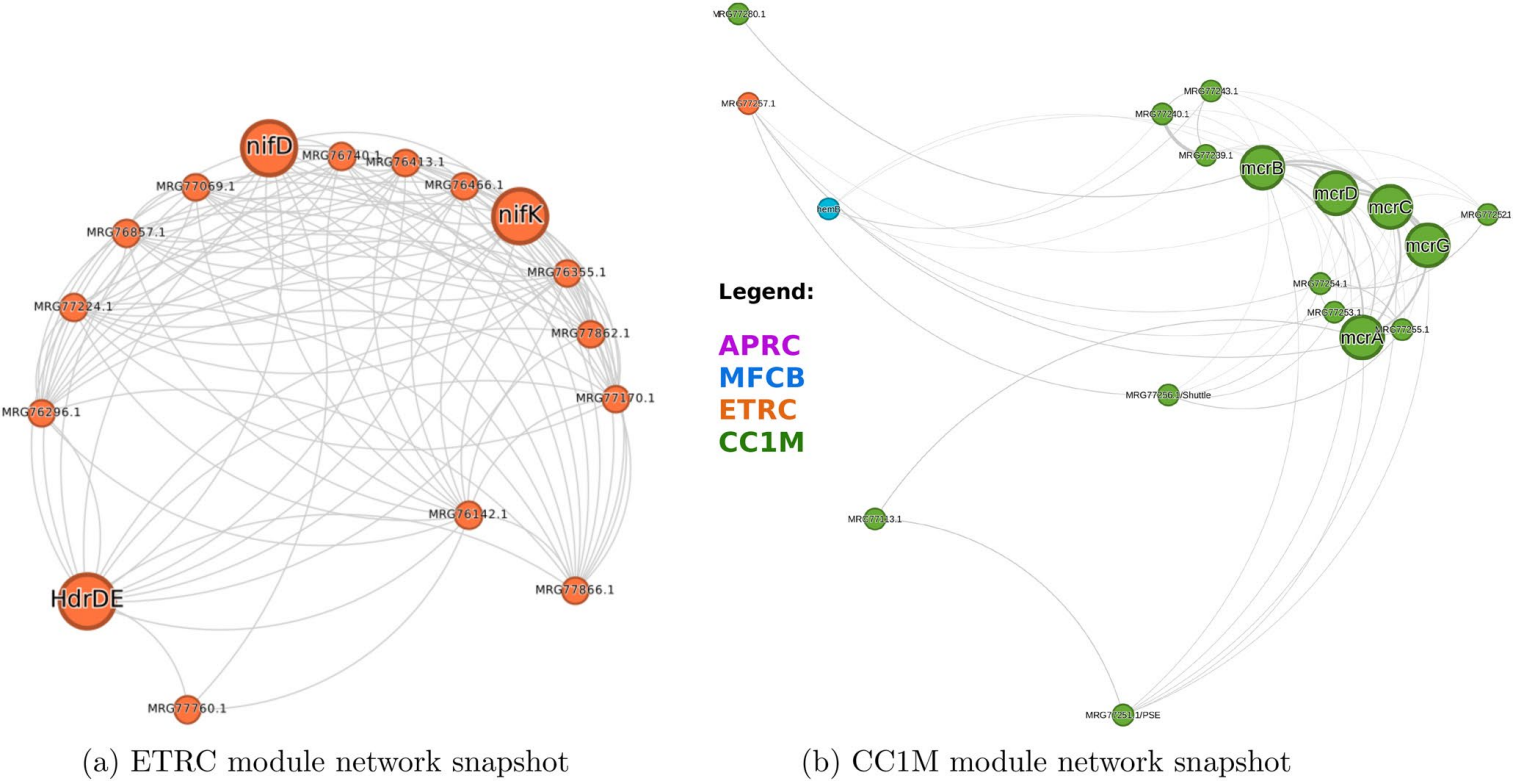
Focused network snapshots from the AOM net highlighting key functional modules. (a) The Electron Transport and Regulatory Core (ETRC) module shows the direct network connection between core nitrogenase components (NifD and NifK) and the AOM electron transfer protein HdrDE, illustrating network‐level evidence for the integration of diazotrophy within the ETRC module. (b) The Central C1 Metabolism (CC1M) module visualises interactions between the five subunits of the Methyl‐Coenzyme M Reductase complex (McrA, B, C, D, G) and its dedicated F430 cofactor shuttle protein, highlighting the modular ‘factory‐delivery‐client’ architecture. Node colours represent module assignments with clear legends mapping each colour to its corresponding module name (APCR, MFCB, ETRC and CC1M). Font sizes for node labels have been increased and edge density reduced to show only the highest‐confidence interactions for improved readability.

Additionally, we performed homology searches using BLASTp to identify components of the Factor F430 biosynthesis (Cfb) pathway. We used well‐annotated Cfb enzymes from *Methanosarcina acetivorans* as queries against the ANME‐2a proteome to identify strong candidates for the complete Cfb pathway, including the essential components CfbA, CfbB, CfbC, CfbD and CfbE. Based on predictions using Operon‐mapper (Taboada et al. 2018), we excluded interactions solely due to operon co‐localisation without additional phylogenetic profile support (12 proteins filtered), as requiring both lines of evidence provides stronger support for a true functional linkage. The final AOM net contains 474 proteins and 19,315 edges (Figure 2).

### 2.4 Network Clustering, Functional Annotation and Pathway Enrichment Analysis

We clustered the AOM net into functional modules using the Louvain method (Blondel et al. 2008) in Gephi (v0.10.1). Our goal was to partition the network into the smallest number of large, functionally coherent modules. Therefore, we used an empirical approach to select the resolution parameter rather than strictly maximising the modularity score. We systematically tested a range of resolution values and selected a value of 0.95, as it was the lowest value that consistently yielded a small number of large, functionally coherent modules. The resulting modularity score was 0.146.

We performed functional annotation of all ANME‐2a proteins using InterProScan (v5.52–86.0) (Blum et al. 2021) against all member databases. We specifically extracted Enzyme Commission (EC) numbers from eggNOG‐mapper v2 annotations performed on the ANME‐2a proteome (Cantalapiedra et al. 2021). We derived MetaCyc pathway annotations from the InterProScan results by parsing the final column of the TSV output.

We conducted pathway enrichment analysis for each AOM net module using a custom Python script (available in Supplementary Materials) implementing the one‐tailed hypergeometric test (scipy.stats.hypergeom.sf function [Virtanen et al. 2020]) to determine if specific MetaCyc pathways were overrepresented in a module compared to the entire ANME‐2a proteome (*N* = 1887 proteins used as background). Our script parsed the InterProScan output to map proteins to pathways. For each pathway and module, the stable number of communities (four) effectively separates core functional systems without over‐fragmenting the network. This resulted in a final modularity score of 0.146 and produced modules APCR, MFCB, ETRC and CC1M, containing 46, 94, 92 and 242 proteins, respectively. The 34 canonical AOM proteins (Table 1) were distributed among these modules.

**TABLE 1:** Canonical AOM‐related proteins in ANME‐2a, their degree in the AOM net, gene name, description and assigned module within the AOM net.

Protein ID	Degree	Gene	Description	Module
MRG76549.1	125	HdrDE	Disulfide reductase (subunit D, cytoplasm)	ETRC
MRG76152.1	214	MHC	Methanogenesis multiheme c‐type cytochrome	APCR
MRG76147.1	8	Rnf	RnfABCDGE type electron transport complex subunit A	CC1M
MRG76148.1	9	Rnf	RnfABCDGE type electron transport complex subunit E	CC1M
MRG76971.1	29	HdrABC	CoB–CoM heterodisulfide reductase subunit C	ETRC
MRG76959.1	91	Ftr	Formylmethanofuran–tetrahydromethanopterin N‐formyltransferase	CC1M
MRG76344.1	12	HdrABC	CoB–CoM heterodisulfide reductase subunit C	CC1M
MRG77667.1	22	Fmd	Formylmethanofuran dehydrogenase subunit B	CC1M
MRG77327.1	11	Mch	Methenyltetrahydromethanopterin cyclohydrolase	CC1M
MRG76548.1	8	HdrDE	Disulfide reductase (subunit E, membrane)	CC1M
MRG77672.1	18	Mtr	Tetrahydromethanopterin S‐methyltransferase subunit A (pse)	CC1M
MRG76766.1	14	Ftr	Formylmethanofuran–tetrahydromethanopterin N‐formyltransferase	CC1M
MRG76891.1	12	Fpo_Fqo	NAD(P)H‐quinone oxidoreductase subunit 3	CC1M
MRG76972.1	3	HdrABC	CoB–CoM heterodisulfide reductase subunit B	CC1M
MRG76973.1	3	HdrABC	Heterodisulfide reductase, partial	CC1M
MRG77674.1	20	Mtr	Tetrahydromethanopterin S‐methyltransferase subunit C	CC1M
MRG77128.1	13	Mtd	F420‐dependent methylenetetrahydromethanopterin dehydrogenase	CC1M
MRG77178.1	2	Fmd	Formylmethanofuran dehydrogenase subunit B, partial	CC1M
MRG77312.1	16	Mtr	Tetrahydromethanopterin S‐methyltransferase subunit H	CC1M
MRG77313.1	15	Mtr	Tetrahydromethanopterin S‐methyltransferase subunit A (cytoplasm)	CC1M
MRG77320.1	10	Mer	5,10‐methylenetetrahydromethanopterin reductase	CC1M
MRG77471.1	10	Fmd	Formylmethanofuran dehydrogenase	CC1M
MRG77666.1	16	Fmd	Formylmethanofuran dehydrogenase	CC1M
MRG77669.1	15	Mtr	Tetrahydromethanopterin S‐methyltransferase subunit H	CC1M
MRG77670.1	15	Mtr	Tetrahydromethanopterin S‐methyltransferase subunit G	CC1M
MRG77671.1	16	Mtr	Tetrahydromethanopterin S‐methyltransferase subunit F	CC1M
MRG77673.1	18	Mtr	Tetrahydromethanopterin S‐methyltransferase subunit B	CC1M
MRG77675.1	19	Mtr	Tetrahydromethanopterin S‐methyltransferase subunit D	CC1M
MRG77676.1	16	Mtr	Tetrahydromethanopterin S‐methyltransferase subunit E	CC1M
MRG77250.1	8	Mcr	Coenzyme‐B sulfoethylthiotransferase subunit alpha (McrA)	CC1M
MRG77246.1	15	Mcr	Coenzyme‐B sulfoethylthiotransferase subunit beta (McrB)	CC1M
MRG77249.1	9	Mcr	Coenzyme‐B sulfoethylthiotransferase subunit gamma (McrG)	CC1M
MRG77248.1	11	Mcr	Methyl‐coenzyme M reductase I operon protein C (McrC)	CC1M
MRG77247.1	15	Mcr	Methyl‐coenzyme M reductase operon protein D (McrD)	CC1M

Functional annotation of all ANME‐2a proteins utilised InterProScan (v5.52–86.0) (Blum et al. 2021) against all member databases. EC numbers were specifically extracted from eggNOG‐mapper v2 annotations performed on the ANME‐2a proteome (Cantalapiedra et al. 2021). MetaCyc pathway annotations were derived from the InterProScan results by parsing the final column of the TSV output.

Pathway enrichment analysis for each AOM net module employed a custom Python script (available in Supplementary Materials) implementing the one‐tailed hypergeometric test (scipy.stats.hypergeom.sf function [Virtanen et al. 2020]) to determine if specific MetaCyc pathways were overrepresented in a module compared to the entire ANME‐2a proteome (*N* = 1887 proteins used as background). The script parsed the InterProScan output to map proteins to pathways. For each pathway and module, the test used the following counts: number of module proteins annotated to the pathway (*k*), total number of proteins in the module (*M*), total number of proteins annotated to the pathway (*K*) and total number of background proteins (*N*). Calculated *p* values were corrected for multiple testing across all tested pathways using the Benjamini‐Hochberg False Discovery Rate (FDR) method (statsmodels.stats.multitest.multipletests function [Benjamini and Hochberg 1995, Seabold and Perktold 2010]). Pathways with an FDR < 0.05 were considered significantly enriched.

To assess pathway completeness for selected pathways of interest (Nitrogen fixation, Mevalonate/Lipid biosynthesis, F420 biosynthesis), the list of essential catalytic enzyme EC numbers predicted for the ANME‐2a proteome by eggNOG‐mapper (Cantalapiedra et al. 2021) was compared. The modular localisation of identified key enzymes was then checked against the module protein lists.

Manual curation of selected protein interactions within the AOM net involved consulting annotation databases (NCBI, InterPro, Pfam) and relevant literature for proteins identified as neighbours to core AOM components, generating specific functional hypotheses (see Supplementary Material B for details). Bridging centrality calculations for the AOM net utilised network analysis tools to pinpoint key integrating proteins for further investigation (see Supplementary Material C for details).

## 3 Results

### 3.1 PPI Network and AOM Subnetwork Construction

Using a comparative genomic approach across 230 genomes from the order Methanosarcinales—the closest phylogenetic relatives to ANME‐2a—GenPPi inferred a comprehensive PPI network for the target ANME‐2a isolate. This feature‐based network comprises 1869 protein nodes, representing 99% of the predicted proteome, connected by 43,443 predicted interactions (edges). Neighbourhood conservation supported 8767 interactions, while conserved phylogenetic profiles—the shared presence or absence of proteins across a set of genomes—across the analysed genomes accounted for 35,028 interactions. The network's degree distribution exhibited power‐law characteristics typical of biological networks (see Supplementary Material A and Figure 3).

To focus on processes directly linked to anaerobic methane oxidation, we constructed a subnetwork referred to as the AOM net. This subnetwork included the 34 protein products of the 12 canonical AOM‐related genes (Fmd, Fpo/Fqo, Ftr, HdrABC, HdrDE, Mch, Mcr, Mer, MHC, Mtd, Mtr and Rnf) plus their immediate neighbours in the full PPI network (total 474 proteins). Interactions based solely on operon proximity without additional phylogenetic support were excluded (12 proteins filtered from the initial neighbour set). The resulting AOM net is a densely connected subgraph, indicating a significant portion of the ANME‐2a interactome is closely linked to the core AOM machinery (Figure 2).

### 3.2 Modular Structure Within the AOM Net and Statistical Pathway Enrichment

To investigate the functional organisation within the AOM net, we applied the Louvain clustering algorithm (Blondel et al. 2008). This method partitions the network into modules by grouping proteins that are more densely connected than the rest of the network, suggesting they form distinct functional units. Empirical adjustment of the resolution parameter yielded four distinct groups or modules within this subnetwork, whose functions were inferred from their protein composition and pathway enrichment analysis:

- Antibiotic Production and Cofactor Recycling (APCR): 46 proteins, a module proposed to function as an extracellular redox interface. While not significantly enriched for any single metabolic pathway in our analysis, it contains the canonical multiheme c‐type cytochrome (MHC), a key component implicated in extracellular electron transfer.
- Membrane Fortification and Central Biosynthesis (MFCB): 92 proteins, a super‐module acting as the primary hub for structural integrity and peripheral metabolism, significantly enriched for pathways involved in archaeal lipid biosynthesis (Membrane Fortification).
- Electron Transport and Regulatory Core (ETRC): 94 proteins, a module functioning as a bioenergetic interface and the nexus for diazotrophy. It is centred on key Electron Transport components and co‐clusters of AOM electron transfer proteins (Hdr) with the core nitrogenase subunits (NifD/K) within the network's modularity, not its chromosomal location.
- Central C1 Metabolism (CC1M): 242 proteins, representing the stable enzymatic engine of the network. This module contains the vast majority of the canonical AOM pathway enzymes responsible for the core catalytic steps of Central Carbon (C1) Metabolism.

The distribution of the 34 canonical AOM proteins across these modules is detailed in Table 1. This distribution underscores that the modules are not merely peripheral but incorporate distinct and complementary aspects of the core AOM functionality.

Pathway enrichment analysis utilised a hypergeometric test followed by the Benjamini‐Hochberg correction for FDR to identify potential functional specialisations within these AOM net modules. Enrichment testing of MetaCyc pathways within each module's protein set was performed against the background of all proteins in the ANME‐2a genome (*N* = 1887) based on annotations derived from InterProScan (see Methods).

This analysis revealed distinct enrichment profiles for the module MFCB exhibiting significant enrichment (FDR < 0.05) for pathways related to archaeal lipid biosynthesis, supporting its role in membrane fortification. No other modules showed significant enrichment for the pathways tested. Table 2 presents a sample of significantly enriched pathways for the MFCB module.

**TABLE 2:** Selected significantly enriched MetaCyc pathways (FDR < 0.05) identified through statistical pathway enrichment analysis.

Pathway name (representative/known)	FDR
Archaeal lipid biosynthesis (general variant)	< 0.05
Mevalonate pathway I	< 0.05
Adenosylcobinamide‐GDP salvage	< 0.05
Paromomycin biosynthesis	< 0.05
Salinosporamide A biosynthesis	< 0.05
UTP and CTP dephosphorylation I	< 0.05

Our initial pathway enrichment analysis identified 411 significantly enriched MetaCyc pathways within the MFCB module (FDR < 0.05), confirming its high level of metabolic activity. While statistically robust, this large number of overlapping pathways made it challenging to distill the module's core functional identity. To achieve a higher functional resolution and move from broad statistical inference to a direct inventory of molecular functions, we adopted a complementary ‘bottom‐up’ analytical approach.

We performed a functional annotation for all 92 proteins in the MFCB module to identify their specific KEGG Orthology (KO) identifiers. This analysis revealed that 44 out of 92 proteins correspond to 49 unique KO terms (see Appendix E, Table 8 for a complete list of protein‐KO assignments). The apparent reduction from 411 enriched pathways to 49 unique functions is an expected and informative consequence of this change in analytical resolution. A single, metabolically versatile enzyme (represented by a single KO term) can be a constituent of dozens of interconnected metabolic pathways. The initial enrichment analysis counted all these redundant pathways, whereas the KO‐based approach provides a nonredundant inventory of the module's specific functional ‘parts list’. This more direct functional profile allowed us to confidently categorise the module's primary roles into the clear themes presented in Table 3, providing a more robust foundation for our conclusions.

**TABLE 3:** Key functional categories enriched within the MFCB module, based on KEGG Orthology (KO) analysis.

Functional category	Description and relevance to the MFCB module	No. of KOs
Central metabolism and energy	Represents the module's energetic and anabolic engine, including foundational pathways like Carbon Metabolism, the TCA Cycle and Oxidative Phosphorylation.	20
Biosynthesis (essential components)	Production of cellular building blocks, such as secondary metabolites, cofactors (vitamins) and amino acids, essential for growth and maintenance.	13
Membrane fortification	Provides key biochemical evidence for the membrane reinforcement hypothesis via the terpenoid‐quinone biosynthesis pathway, direct precursors to hopanoids.	1
Environmental interaction	Includes systems for environmental sensing and response, such as Quorum Sensing and Two‐Component Systems, indicating a role in intercellular communication and adaptation.	2
Methane oxidation	No canonical pathways associated with this function were found to be enriched within the module, suggesting this machinery, if present, is organised elsewhere in the interactome.	0
Nitrogen fixation	No canonical pathways associated with this function were found to be enriched within the module, consistent with the absence of *nif* genes in this protein subset.	0

The results confirmed that MFCB is overwhelmingly dominated by proteins involved in central metabolism and energy generation, as well as the biosynthesis of essential cellular components. Crucially, this detailed KO‐based analysis provided direct evidence supporting our membrane fortification hypothesis through the identification of the ‘Ubiquinone and other terpenoid‐quinone biosynthesis’ pathway. Terpenoids are the direct biochemical precursors to hopanoids, which are archaeal lipids known to reinforce membrane structure. In contrast, our comprehensive search for key functional markers within the enriched functions of the MFCB module found no evidence for pathways related to methane oxidation or nitrogen fixation. It is important to emphasize that MFCB was the only module out of the four (APCR, MFCB, ETRC and CC1M) to show any statistically significant pathway enrichment, highlighting its unique role as a dense and functionally specialised hub within the AOM‐associated network.

### 3.3 Assessment of Key Pathway Completeness and Modular Localisation

To complement the enrichment analysis and assess functional potential, we evaluated the presence of key catalytic enzymes for selected pathways of interest within the ANME‐2a genome, identified by EC numbers via eggNOG‐mapper (Cantalapiedra et al. 2021) (see Methods). We determined their distribution across the AOM net modules.

Nitrogen Fixation (Nif Pathway): Building upon previous reports suggesting diazotrophy based on nifH expression (Metcalfe et al. 2021), our genome‐wide annotation confirmed the presence of a comprehensive and complete set of genes essential for nitrogen fixation in ANME‐2a (Table 4). We identified genes encoding the core nitrogenase components NifH (MRG76467.1, EC 1.18.6.1), NifD (MRG76470.1, EC 1.18.6.1) and NifK (MRG76471.1, EC 1.18.6.1), along with the complete set of essential FeMo‐cofactor biosynthesis proteins, including the critical Radical SAM enzyme NifB (MRG77339.1), NifE (MRG76472.1) and NifN (MRG76473.1) and other biosynthesis proteins and P‐II regulators (e.g., MRG77531.1, MRG76468.1). Within the AOM net modules, the core nitrogenase subunits NifD and NifK were located in module ETRC; the remaining Nif components reside outside these immediate AOM‐neighbour modules.

**TABLE 4:** Nitrogen fixation‐related proteins identified in the ANME‐2a genome (GCA_009649835.1) and their assigned EC numbers and AOM net module localisation.

Protein ID	Putative function	EC number	Module assignment
MRG77339.1	Nitrogenase iron‐molybdenum cofactor biosynthesis protein (NifB)	—	
MRG77494.1	Dinitrogenase iron‐molybdenum cofactor biosynthesis protein	—	
MRG77256.1	Dinitrogenase iron‐molybdenum cofactor biosynthesis protein	—	
MRG76471.1	Nitrogenase molybdenum‐iron protein subunit beta (NifK)	1.18.6.1	ETRC
MRG76472.1	Nitrogenase iron‐molybdenum cofactor biosynthesis protein NifE	—	
MRG77496.1	Dinitrogenase iron‐molybdenum cofactor biosynthesis protein	—	
MRG76467.1	Nitrogenase iron protein (NifH)	1.18.6.1	
MRG76473.1	Nitrogenase‐associated protein N (NifN)	—	
MRG76470.1	Nitrogenase molybdenum‐iron protein alpha chain (NifD)	1.18.6.1	ETRC
MRG77531.1	P‐II family nitrogen regulator	—	
MRG76469.1	P‐II family nitrogen regulator	—	
MRG76468.1	P‐II family nitrogen regulator	—	

*Note:* All listed proteins were predicted to be cytoplasmic. EC numbers are based on Cantalapiedra et al. (2021).

Mevalonate Pathway and Archaeal Lipid Biosynthesis (e.g., PWY‐7321): Our statistical enrichment analysis highlighted module MFCB as significantly enriched (FDR < 0.05) for the Mevalonate pathway I (PWY‐7321) and the general Archaeal lipid biosynthesis pathway (PWY‐7771), among others (Table 2). Assessment based on key EC numbers confirmed the presence of genes encoding upstream enzymes for the mevalonate pathway in the ANME‐2a genome, such as HMG‐CoA synthase (EC 2.3.3.10, MRG77149.1), HMG‐CoA reductase (EC 1.1.1.34, MRG77042.1) and Mevalonate kinase (EC 2.7.1.36, MRG76652.1). However, consistent with their role in general biosynthesis, our analysis found these specific core enzymes outside the defined AOM net modules. Clear homologues for enzymes catalysing later steps, like Phosphomevalonate kinase (EC 2.7.4.2) and Diphosphomevalonate decarboxylase (EC 4.1.1.33), were not found via EC number mapping, potentially due to the draft genome status or annotation divergence. Nonetheless, the enrichment of associated pathways in module MFCB suggests that components involved in the modification, regulation or downstream utilisation of isoprenoids are concentrated within this module. This underscores the importance of these archaeal‐specific isoprenoid lipids for membrane structure and function in ANME‐2a (Jain et al. 2014).

Coenzyme F420 Biosynthesis (PWY‐6409): This pathway appears largely complete based on our genome‐wide annotation. Key enzymes were identified based on EC numbers, including homologues for FbiB (e.g., EC 6.3.5.10, MRG76395.1), FbiC (e.g., EC 4.1.99.22, MRG76226.1) and components related to F420‐dependent enzymes (e.g., EC 1.5.99.15, MRG76852.1). This pathway did not reach statistical significance (FDR < 0.05) in our formal enrichment analysis for any specific module. However, several associated enzymes, particularly MRG76395.1 (FbiB) and MRG76852.1 (F420‐dependent oxidoreductase), were located within module CC1M in the AOM net clustering, which also contains a majority of the canonical AOM enzymes, including those directly utilising F420 like Mtd and Mer (Table 1). This co‐localisation suggests a tight functional integration of F420 metabolism with the core AOM processes within module CC1M, consistent with the known essentiality of F420 in methanogenesis and related archaeal energy metabolisms (Thauer 2011, Laso‐Perez et al. 2016).

Antibiotic Biosynthesis Pathways (e.g., PWY‐7018): While the genome of the ANME‐2a isolate contains genes associated with antibiotic biosynthesis pathways, our analysis did not find these pathways to be significantly enriched within any specific functional module. This suggests that while the capacity for producing antimicrobial compounds may exist, it is not concentrated within a single, functionally cohesive unit of the AOM‐associated network.

### 3.4 The MCR and F430 Biosynthesis Pathways Form Distinct Functional Modules

Our analysis revealed that the core subunits of the MCR complex were indeed present in our full genome‐wide PPI network and correctly predicted their interactions with essential support proteins (Table 6). The network identified a sophisticated functional module centred around MCR that includes cofactor delivery, metal transport, transcriptional regulation and assembly machinery.

Further investigation of the MCR genomic neighbourhood revealed an extended functional network of proteins that contribute to the MCR module's operation (Table 6). This extended analysis uncovered critical components for cofactor biosynthesis, including a porphobilinogen synthase (MRG77243.1, EC 4.2.1.24) essential for tetrapyrrole precursor synthesis, and multiple iron–sulfur cluster proteins (MRG77252.1, MRG77254.1) that likely participate in the complex redox chemistry associated with MCR function. Additionally, we identified energy‐coupling components such as a P‐loop NTPase (MRG77257.1) and regulatory elements, including a cytidine deaminase (MRG77239.1, EC 3.5.4.12), suggesting sophisticated posttranscriptional control mechanisms.

Through a combination of genome annotation and targeted BLASTp homology searches using enzymes from *M. acetivorans* as queries, we successfully identified a complete genetic toolkit for the Factor F430 biosynthesis (Cfb) pathway in ANME‐2a (Table 5). Our analysis located clear orthologs for all essential components, including CfbA, CfbC, CfbD and the crucial coenzyme F430 synthase, CfbE (MRG77108.1). The homology searches yielded exceptionally strong matches with highly significant e‐values (e.g., CfbE: e‐value 1.10e‐117), confirming that this isolate possesses the full genomic blueprint for a functional Cfb pathway. Analysis of genomic locations revealed that the identified Cfb genes are clustered together, forming a likely Cfb operon. Crucially, the gene for the putative F430 shuttle protein belonging to the NifX family (MRG77256.1) is located distantly from this operon.

**TABLE 5:** The modular organisation of the Factor F430 biosynthesis (Cfb) pathway and its delivery bridge in ANME‐2a. Genomic data reveal a core Cfb operon and distinct, individually located genes for a putative shared enzyme (CfbB) and the MCR‐interacting linker protein. Homology data from BLASTp searches using *Methanosarcina acetivorans* Cfb proteins as queries.

Module/component	Protein ID	Genomic locus	Query size (aa)	Homology data (identity%/E‐value)
Core Cfb operon				
CfbA	MRG77107.1	05005	130	57.3%/1.66e‐52
CfbE	MRG77108.1	05010	471	45.9%/1.10e‐117
CfbD	MRG77109.1	05015	370	74.9%/0.0
CfbC	MRG77110.1	05020	265	68.1%/1.47e‐130
Distant component				
CfbB‐like	MRG76925.1	04080	497	40.2%/1.57e‐98^a^
Delivery protein				
F430 Shuttle Protein (NifX family)	MRG77256.1	05775	206	Linker protein^b^

^a^ The distant genomic location and moderate identity suggest this CfbB homologue may be a shared enzyme involved in multiple cofactor biosynthesis pathways.

^b^ This protein is genomically distant from the Cfb operon. Its predicted interaction with the MCR complex, but not with the Cfb operon, supports its role as a dedicated F430 shuttle, a paralog to the NifX proteins that serve the nitrogenase system.

This genomic separation provides a clear rationale for why our PPI network, which relies on conserved gene neighbourhoods as a primary source of evidence, did not predict interactions between the Cfb operon and the F430 Shuttle Protein (NifX family MRG77256.1). The network correctly identified two separate functional modules: the biosynthesis module (Cfb operon) and the utilisation module (MCR complex), connected by a specific protein that appears to mediate the delivery of the cofactor. This modular organisation supports a sophisticated biological model where the Cfb pathway functions as an autonomous metabolon, with the bridge protein serving as a dedicated delivery system to the MCR complex.

## 4 Discussion

Combining genome‐wide PPI network inference, modularity analysis, statistical pathway enrichment, pathway completeness assessment and manual curation, this study explored the metabolic capabilities and functional organisation surrounding the core AOM machinery in ANME‐2a. Our results reveal that the AOM process in this isolate is supported by a modularly organised network that connects the core enzymatic machinery to distinct modules responsible for diazotrophy, membrane fortification and ecological competition.

### 4.1 A Modular Blueprint for Metabolic Integration

A key discovery of our network analysis is the functional compartmentalisation of the AOM‐associated proteome into distinct modules, each with a specialised role. The stable enzymatic engine of AOM is housed within the CC1M module, which contains the vast majority of canonical AOM pathway enzymes (Table 1). This core engine is functionally integrated with three other modules that provide structural, biosynthetic and ecological support.

The MFCB module is dedicated to maintaining the cell's structural integrity, showing significant enrichment for archaeal lipid biosynthesis pathways (Table 2). This ‘membrane fortification’ addresses a critical challenge for ANME organisms: maintaining membrane integrity under the electron‐rich conditions generated by AOM. The significant enrichment for isoprenoid and lipid biosynthesis pathways suggests that this isolate has evolved specialised mechanisms to modify its membrane composition in response to the unique bioenergetic demands of methane oxidation. Such structural reinforcement is likely a key evolutionary adaptation to the extreme physical and chemical conditions of deep‐sea methane seeps, which include high hydrostatic pressure and steep ionic gradients that challenge membrane integrity.

Specific intermodule interactions exemplify this integration between structural support and core bioenergetics. For instance, the hypothetical protein MRG76144.1, a component of the MFCB module, possesses a Universal Stress Protein (USP) domain. Our network predicts its interaction with the Rnf complex (housed in the CC1M module), which interconverts ion gradients and oxidised/reduced electron carriers. This specific link suggests a potential mechanism for coordinating the cell's response to osmotic or ionic stress, a critical function for membrane integrity, directly with the energetic state of the core AOM pathway.

The APCR module is hypothesised to act as a key interface for extracellular redox processes. Although not statistically enriched for specific biosynthetic pathways, its most prominent canonical component is the MHC. The MHC is a critical protein for electron transfer, potentially mediating interactions with syntrophic partners or alternative electron acceptors. Therefore, the APCR module may group proteins responsible for sensing the extracellular environment and managing electron flow at the cell periphery.

### 4.2 Network‐Level Evidence for the Integration of AOM and Diazotrophy

Our analysis provides strong network‐level evidence for the energetic coupling of AOM and nitrogen fixation. The core catalytic components of nitrogen fixation, NifD and NifK, which form the dinitrogenase where N_2_ reduction occurs, were co‐clustered in the ETRC module. Crucially, this module also contains key electron‐transferring components of the AOM pathway, specifically subunits of the Heterodisulfide Reductase (Hdr) complex (Table 1; Figure 2a). This co‐clustering suggests a direct functional and energetic coupling, where the core nitrogen fixation machinery is linked to its primary source of low‐potential electrons generated by AOM. It is important to clarify that this predicted coupling represents a dominant functional and co‐evolutionary association inferred from genomic context, rather than a confirmed direct physical interaction. The network highlights the fundamental linkage between the ‘power plant’ (AOM machinery) and the ‘factory’ (nitrogenase), providing a powerful hypothesis for a direct bioenergetic connection.

This network‐level evidence addresses a fundamental question in ANME ecology: how do these organisms energetically support the ATP‐expensive process of nitrogen fixation while performing AOM? This metabolic capability is of significant ecological importance, as the nitrogen‐limited nature of many methane seep environments places a strong selective pressure on organisms that can perform in situ nitrogen fixation to support biomass synthesis (Strous et al. 2006; Nawaz et al. 2025). Our analysis suggests that the low‐potential electrons generated during methane oxidation are directly channeled to the nitrogenase complex via the Hdr system, creating an efficient metabolic coupling that allows this ANME‐2a isolate to function as an autonomous diazotroph. The genomic confirmation of a complete nitrogen fixation toolkit (Table 4) provides robust evidence for this capability. However, our comparative analysis of other available draft genomes suggests that this diazotrophic potential may not be a universal trait among all ANME‐2a lineages.

The discovery of specialised NifX‐family shuttle proteins provides additional evidence for sophisticated metabolic cross‐talk management. Our analysis identified paralogous shuttle proteins: one set (MRG77494.1/MRG77496.1) located near the Nif operon for cofactor delivery to nitrogenase, and another (MRG77256.1) dedicated exclusively to F430 cofactor delivery to the MCR complex (Figure 2b). This elegant evolutionary strategy maintains modular separation of cofactor delivery systems while enabling energetic integration, exemplifying the sophisticated biological engineering underlying AOM‐diazotrophy coupling.

### 4.3 Implications for Archaeal Adaptation and Metabolic Evolution

These findings have broader implications for understanding archaeal adaptation to electron‐rich environments and the evolution of metabolic coupling systems. The membrane fortification module suggests that ANME organisms have evolved beyond simple metabolic versatility to achieve sophisticated metabolic integration, where the primary energy‐generating process directly supports the cellular infrastructure required for that process. This represents a form of metabolic self‐organisation that may be characteristic of organisms adapted to extreme or specialised ecological niches.

The direct coupling of AOM with nitrogen fixation provides a model for how energetically expensive biosynthetic processes can be supported in environments where traditional electron acceptors are limited. This coupling strategy may explain how ANME organisms can thrive as autonomous diazotrophs in methane‐rich environments, contributing both to carbon cycling through methane oxidation and to nitrogen cycling through nitrogen fixation (Rahman et al. 2025).

Our comparative genomic analysis reveals that while nitrogen fixation capability varies among ANME‐2a lineages, the membrane fortification pathways are highly conserved, suggesting they represent fundamental adaptive traits for the clade. This conservation pattern indicates that membrane fortification is a core requirement for the ANME‐2a lifestyle, while diazotrophy provides a specialised advantage in nitrogen‐limited environments.

### 4.4 Contextualising Findings With Recent ANME Studies

Our network predictions align well with recent experimental studies on ANME metabolism. The central role of multiheme c‐type cytochromes predicted by our network is supported by studies on ANME‐2a performing iron‐dependent AOM (Slobodkin et al. 2023) and *Candidatus Methanoperedens* performing manganese‐dependent AOM (Leu et al. 2020), both of which revealed extensive MHC expression. Our network extends these findings by proposing a specific functional context for the MHC within a dedicated module (APCR), potentially linking extracellular electron transfer to other processes at the cell's periphery.

While previous studies focused primarily on electron‐accepting pathways for metal reduction, our network provides novel, testable hypotheses for how ANME organisms support their biosynthetic needs. The identification of integrated modules for membrane fortification (MFCB) and nitrogen fixation (ETRC), coupled to the core AOM engine (CC1M), proposes specific mechanisms for how this isolate achieves metabolic autonomy in its ecological niche.

### 4.5 The Modular Architecture for MCR Function and Cofactor Supply

A particularly significant discovery from our analysis is the identification of an extensive network of proteins that interact with and support the MCR complex—the enzyme responsible for the first and most critical step in AOM: methane activation. Our network analysis revealed a rich functional module centred around MCR, whose key supporting components are detailed in Table 6. This comprehensive analysis demonstrates that MCR function is supported by a sophisticated modular architecture encompassing three key organisational levels: the cofactor biosynthesis ‘factory’, the delivery system, and the target enzyme with its regulatory apparatus.

**TABLE 6:** Comprehensive analysis of proteins interacting with the MCR complex: Key enzymes and regulatory proteins identified in the AOM network that support methyl‐coenzyme M reductase function, excluding MCR subunits themselves. Data derived from database queries and network analysis.

Locus tag	Product	Gene	EC number	Subcellular location
Cofactor biosynthesis and delivery				
C5S33_05775	Dinitrogenase iron‐molybdenum cofactor biosynthesis protein	—	—	Cytoplasmic
C5S33_05710	Porphobilinogen synthase	hemB	4.2.1.24	Cytoplasmic
C5S33_05035	Aminotransferase class I/II‐fold pyridoxal phosphate‐dependent enzyme	—	4.1.1.81	Cytoplasmic
Metal transport and ion homeostasis				
C5S33_05750	Cation diffusion facilitator family transporter	—	—	Potentially surface‐Exposed
Electron transfer and redox chemistry				
C5S33_05755	4Fe‐4S dicluster domain‐containing protein	—	—	Cytoplasmic
C5S33_05765	4Fe‐4S dicluster domain‐containing protein	—	—	Cytoplasmic
Energy coupling and nucleotide metabolism				
C5S33_05780	P‐loop NTPase	—	—	Cytoplasmic
C5S33_05690	Cytidine deaminase	—	3.5.4.12	Cytoplasmic
Regulation and assembly				
C5S33_05695	Lrp/AsnC family transcriptional regulator	—	—	Cytoplasmic
C5S33_05900	Proteasome assembly chaperone family protein	—	—	Cytoplasmic
Structural and unknown function				
C5S33_05715	Hypothetical protein	—	—	Cytoplasmic
C5S33_05760	Hypothetical protein	—	—	Cytoplasmic
C5S33_05770	Hypothetical protein	—	—	Cytoplasmic

*Note:* This table excludes MCR subunits (McrA, McrB, McrG, McrC, McrD) and focuses on supporting proteins that interact with or regulate the MCR complex. Functional categories are based on protein domain analysis and literature curation.

This discovery of specialised delivery proteins for distinct metalloenzymes reveals an elegant evolutionary strategy in this ANME‐2a isolate. Our analysis identified two sets of NifX‐family shuttle proteins: one set (MRG77494.1/MRG77496.1) is located near the Nif operon. In contrast, the F430 shuttle (MRG77256.1) is genomically distant, and its network interactions are exclusive to the MCR complex. This insight points to a classic case of molecular adaptation, where paralogous shuttle proteins have been specialised to support the two cornerstone metabolic processes in ANME‐2a. This duplication and specialisation allow the organism to couple these fundamental pathways energetically; this isolate can fuel the immense energetic cost of nitrogen fixation directly with the energy derived from methane oxidation. Furthermore, having dedicated shuttle proteins prevents metabolic cross‐talk, ensuring that the correct, complex metallo‐cofactor is delivered with high fidelity to its specific target enzyme. This finding provides a powerful, molecular‐level example of the modularity we propose throughout this paper, where metabolic pathways are integrated into the cell's overall strategy. However, their component delivery systems are kept independent to ensure efficiency and accuracy.

The modular organisation follows a factory‐delivery‐client model that exemplifies the sophisticated biological engineering underlying AOM. The F430 cofactor biosynthesis pathway operates as an autonomous ‘factory’ organised as a genomically clustered operon (CfbA, CfbC, CfbD), while a dedicated delivery protein (C5S33_05775) serves as the ‘delivery truck’ that shuttles the completed cofactor to the MCR complex (Table 5). This genomic separation between biosynthesis and utilisation modules provides compelling evidence for a sophisticated delivery mechanism that explains why our PPI network, which relies heavily on conserved gene neighbourhoods, correctly identified the delivery protein as the critical link while not predicting direct interactions between the Cfb operon and MCR complex.

As the enzyme catalysing the initial methane activation step, MCR represents the gateway reaction for the entire AOM process, making its proper function and regulation critical for this isolate's ecological role as a methane oxidiser. The supporting apparatus can be functionally categorised into several key systems (Table 6). The cofactor biosynthesis and delivery machinery represents the most critical support system, with the dinitrogenase iron‐molybdenum cofactor biosynthesis protein (C5S33_05775) serving as the essential linker between F430 production and MCR assembly. This protein's dual homology to both nitrogenase cofactor biosynthesis and the Cfb pathway highlights the evolutionary relationship between these complex metalloenzyme systems. It suggests a conserved mechanism for delivering complex organometallic cofactors to their target enzymes.

The metal transport and ion homeostasis component, represented by the cation diffusion facilitator family transporter (C5S33_05750), is particularly significant given the strict nickel requirements for F430 biosynthesis. As a membrane‐associated protein, this transporter is positioned at the interface between the cell and its environment. Its function is likely critical for the uptake of nickel from the surroundings and its delivery to the cytoplasmic cofactor biosynthesis machinery, representing a potential bottleneck for MCR activity under nickel‐limited conditions. The presence of multiple 4Fe‐4S cluster proteins (C5S33_05755, C5S33_05765) within the MCR neighbourhood indicates that electron transfer processes are tightly integrated with the primary catalytic function, likely facilitating the complex redox chemistry required for methane activation and the subsequent electron transfer to downstream acceptors.

The regulatory architecture surrounding MCR is equally sophisticated, with the Lrp/AsnC family transcriptional regulator (C5S33_05695) suggesting that MCR expression is subject to metabolite‐responsive control. This regulatory protein family is known to respond to amino acid availability and carbon/nitrogen balance, indicating that MCR activity may be coordinated with the overall metabolic state of the cell. The proteasome assembly chaperone family protein (C5S33_05900) provides quality control mechanisms essential for proper MCR folding and assembly, while the P‐loop NTPase (C5S33_05780) likely couples ATP hydrolysis to conformational changes required for MCR activation or cofactor insertion.

The presence of several hypothetical proteins (C5S33_05715, C5S33_05760, C5S33_05770) within this functional network suggests that our understanding of MCR regulation and support mechanisms remains incomplete. These proteins, given their genomic proximity and network connectivity to MCR, likely represent novel regulatory or structural components that warrant further experimental investigation. Their conservation within ANME lineages and their integration into the MCR functional module suggest they play important, albeit currently unknown, roles in methane oxidation.

This discovery also clarifies the functional significance of proteins annotated as ‘dinitrogenase iron‐molybdenum cofactor biosynthesis’ proteins. Our homology analyses revealed that the observed sequence similarities between Cfb and Nif proteins reflect genuine evolutionary relationships in metal‐containing cofactor biosynthesis pathways. The exceptionally strong sequence conservation (68%–75% identity for CfbC/CfbD with e‐values ≤ 1.47e‐130) demonstrates that these pathways share common ancestral origins and biochemical mechanisms. This modular cofactor biosynthesis and delivery system represents a key organisational principle that reinforces our central thesis about metabolic modularity as a fundamental strategy in ANME‐2a.

### 4.6 Genomic Potential for Nitrogen Fixation and Its Network Context

A significant contribution of this study is the confirmation, through detailed genomic annotation and EC number identification, of a complete genetic toolkit for nitrogen fixation resident within the reference ANME‐2a genome (Table 4). This finding provides robust genomic evidence for the potential of this specific ANME‐2a isolate to function as an autonomous diazotroph. It should be noted that nitrogen fixation capability is not universal among ANME‐2a lineages, as genomic surveys reveal variable presence of nitrogenase genes across different isolates (see Appendix D, Table 7). Our network analysis provides insights into the predicted functional integration of diazotrophy with AOM energetics. Most *nif* genes reside outside the AOM net modules. However, the core nitrogenase structural subunits NifD (MRG76470.1) and NifK (MRG76471.1) are located within module ETRC, which also harbours canonical AOM components like Hdr subunits (Table 1). Our feature‐based PPI network predicted interactions between NifD/K (in ETRC) and key AOM electron transport proteins HdrD (MRG76549.1, in ETRC) and MHC (MRG76152.1, in APCR). The denser machine learning‐based network suggested alternative links via Nif support machinery to different AOM core enzymes. Both approaches support functional cross‐talk between these processes in this ANME‐2a isolate.

### 4.7 Integrated View of ANME‐2a Metabolic Versatility and MCR Support Network

Collectively, our integrated analysis presents a picture of this ANME‐2a isolate as an organism with a highly adapted core AOM pathway, whose functionality is supported and extended by a modular network, facilitating extensive metabolic versatility. The AOM pathway itself is modularly distributed. The CC1M module constitutes the stable enzymatic engine. This engine is supported by the MFCB module for structural integrity, the APCR module for ecological competition and efficiency, and the ETRC module, which serves as a critical nexus for integrating AOM energetics with nitrogen fixation. ANME‐2a possesses the complete genomic blueprint for nitrogen fixation. This versatility, organised in a modular fashion and connected through key bridging proteins (detailed in Supplementary Material C), likely enables this isolate to thrive in methane seeps.

The extensive MCR support network identified in our analysis (Table 6) exemplifies the sophisticated regulatory and metabolic integration that characterises this ANME‐2a isolate's adaptation to its ecological niche. The presence of dedicated cofactor biosynthesis machinery, metal transport systems, electron transfer proteins and regulatory elements within the MCR functional module demonstrates that methane oxidation is not simply a core catalytic process, but rather a highly regulated and metabolically integrated system. This integration extends beyond the immediate MCR neighbourhood to include connections with amino acid metabolism (aminotransferase, C5S33_05035), nucleotide regulation (cytidine deaminase, C5S33_05690) and protein quality control (proteasome assembly chaperone, C5S33_05900), indicating that MCR function is coordinated with the broader cellular metabolic state. Such tight integration likely provides this isolate with the flexibility to modulate methane oxidation rates in response to environmental conditions, substrate availability and energy demands, contributing to its ecological success in variable methane seep environments.

### 4.8 Comparative Genomics Reveals Core and Accessory Modules in ANME‐2a

To place findings in a broader context and assess the generality of the observed metabolic capabilities, a comparative genomic analysis of the key functional modules was performed across four other publicly available ANME‐2a genomes. These results were interpreted with caution, considering the varying degrees of completeness of these draft genomes (ranging from 1200 to 2500 predicted proteins, compared to 1887 in our reference genome). A detailed summary of this analysis is presented in Appendix D.

This comparative analysis suggests that diazotrophy is a variable trait within the ANME‐2a clade. Notably, the most complete genome in the set (GCA_013374455) contains the *nifHDKEN* structural gene cluster but lacks a clear ortholog for the essential cofactor biosynthesis enzyme, *nifB*. While this could imply the nitrogen fixation pathway is nonfunctional in this isolate, it is equally possible that the *nifB* gene resides in a gap within the current genome assembly. This ambiguity underscores the challenge of drawing firm conclusions from draft genomes. Similarly, the absence of the entire *nif* cluster in other, more fragmented genomes prevents a definitive assessment. Based on the available evidence, our findings are consistent with a model where the complete system for diazotrophy is an accessory metabolic function. However, confirmation will require more complete genome sequences.

In stark contrast, the core pathways for ‘membrane fortification’ (archaeal lipid biosynthesis) and the representative genes from our identified secondary metabolite cluster were highly conserved across the analysed genomes, even in the more fragmented ones. The consistent presence of these pathways suggests they represent fundamental, core adaptive traits for the ANME‐2a clade. While nitrogen fixation provides a powerful but specialised advantage, a robust and fortified membrane structure, coupled with the potential for producing secondary metabolites for competition or signalling, may be a more universal requirement for thriving in the physically and chemically challenging deep‐sea methane seep environment.

### 4.9 Limitations and Future Perspectives

We emphasise that pathway enrichment and completeness based on genomics require functional validation. A key aspect of our computational approach is the generation of novel interaction predictions not present in curated databases like STRING. These discrepancies should be interpreted not as errors, but as testable hypotheses supported by strong genomic context evidence (conserved gene neighbourhood and phylogenetic profiles). These novel predictions are particularly valuable for generating new insights into the biology of less‐characterised organisms like ANME‐2a and represent prioritised candidates for future experimental validation. We emphasise that pathway enrichment and completeness based on genomics require functional validation. The draft status of the ANME‐2a genome might explain missing enzymes in some pathways (Denton et al. 2014). Importantly, our findings are specific to the ANME‐2a isolate studied. While our comparative genomic survey suggests variability in the distribution of nitrogen fixation genes, we strongly caution against concluding that these genes are definitively absent in other lineages. The draft quality of publicly available genomes means that an unobserved gene may reside in an assembly gap. This is a critical limitation of the current data; distinguishing between true biological absence and technical artefacts will require the generation of complete, closed genomes for multiple ANME‐2a isolates. Therefore, while our data point toward diazotrophy being a variable, strain‐specific adaptation, this remains a hypothesis pending confirmation with higher‐quality genomic resources. Future work involving metatranscriptomics and metaproteomics is needed to confirm the expression and activity of these pathways in situ. Future work should focus on validating these computational predictions. While the significant challenge of cultivating ANME‐2a in the laboratory currently renders experiments requiring native biomass inviable, the key hypotheses generated by our network can be tested directly using well‐established molecular techniques. Priority interactions, such as the coupling between the Nif and Hdr complexes or the delivery of F430 to the MCR complex, can be investigated using in vitro pull‐down assays with recombinant proteins expressed in heterologous hosts like *Escherichia coli*. Furthermore, the yeast two‐hybrid system provides a powerful in vivo platform to screen for these interactions. Concurrently, continuing to probe these ecosystems with metatranscriptomics and metaproteomics will be essential to confirm the expression and activity of these integrated pathways in situ, providing a crucial link between genomic potential and real‐world function.

## 5 Conclusion

By looking beyond the canonical role of anaerobic methane oxidation, this study has decoded the protein landscape of this ANME‐2a isolate, revealing a deeply integrated blueprint for niche adaptation. Our network‐based approach demonstrates that this isolate represents far more than a simple methane oxidiser; it exemplifies a highly autonomous organism whose core energy metabolism is intricately wired to systems for nitrogen fixation and membrane fortification. A key insight is that the AOM pathway is not a monolithic block but is functionally compartmentalised, with its components distributed across distinct modules that are deeply integrated with suites of adaptive functions. This organisation provides a network‐level explanation for the metabolic versatility observed in this ANME‐2a isolate.

This blueprint is built upon specialised modules, each contributing a unique layer to the organism's adaptive metabolism: the CC1M module houses the core AOM enzymatic engine; the MFCB module acts as a major hub for cellular maintenance and membrane fortification; the APCR module, centred on the MHC, likely manages extracellular redox interactions and the ETRC module provides a critical nexus integrating the energetics of AOM with nitrogen fixation. Crucially, our work confirms that this ANME‐2a isolate possesses the complete genomic toolkit for autonomous diazotrophy, representing a potential cornerstone of adaptive strategy in nitrogen‐limited methane seep environments for diazotrophic ANME‐2a lineages.

In summary, our findings depict this ANME‐2a isolate not merely as a versatile microorganism but as an organism equipped with an intricate network architecture that enables robust ecological adaptation. This modular blueprint provides a model for how diazotrophic ANME‐2a lineages may thrive in dynamic ecosystems. A key insight from this blueprint is the discovery of a shared evolutionary design, where paralogous molecular machinery has been specialised to energetically couple the organism's two deeply integrated metabolic pillars: energy‐yielding methane oxidation and energy‐consuming nitrogen fixation. This synergy provides a molecular basis for its ecological success. Identifying the specific hypothetical proteins that form these crucial bridges pinpoints high‐priority targets for future experimental characterisation, which promises further to elucidate the complex biology of this globally significant archaeon.

## Author Contributions

**Samuel de Souza e Silva:** investigation, writing – original draft. **Natanael Borges de Avila:** investigation, writing – original draft. **Lucas Ramos Fernandes da Silva:** investigation. **Matheus Ribeiro Prado:** investigation. **Alisson William da Silva:** investigation. **Murilo Alves Beppler:** validation. **Anderson Rodrigues dos Santos:** conceptualisation, methodology, software, formal analysis, investigation, data curation, writing – original draft, writing – review and editing, supervision. All authors read and approved the final manuscript.

## Conflicts of Interest

The authors declare no conflicts of interest.

## Supporting information


**Data S1:** emi470233‐sup‐0001‐supinfo.zip.

**Appendix S1:** emi470233‐sup‐0002‐AppendixS1.pdf.

## Data Availability

The whole genome shotgun sequence for ANME‐2a (MAG: Candidatus Methanocomedens sp. isolate S7142MS1 2a_fsr_manual, whole genome shotgun sequence) is available in the NCBI GenBank repository under accession number GCA_009649835.1. The complete, unfiltered protein–protein interaction network is publicly available in multiple formats (Gephi, DOT and TSV for use with R/Bioconductor), and other data supporting the findings of this study are openly available in Zenodo (Santos 2025). The GenPPi software is publicly available on GitHub at santosardr/genppi.
