# Supplementary material for: Beyond Methane Oxidation: The Protein Landscape of ANME‐2a Reveals an Integrated System for Diazotrophy and Membrane Fortification
Source: Environ Microbiol Rep. 2025 Nov 17;17(6):e70233. doi: 10.1111/1758-2229.70233 (PMC12622379; doi:10.1111/1758-2229.70233)
Supplement: Supplementary file 1 — Data S1: emi470233‐sup‐0001‐supinfo.zip. [file EMI4-17-e70233-s001.zip › appendix.pdf]

Beyond Methane Oxidation: The Protein Landscape of ANME-2a Reveals an Integrated System for Diazotrophy and Membrane Fortification

Samuel de Souza e Silva[1,†], Natanael Borges de Avila[1,†], Alisson William da Silva[2], Lucas Ramos Fernandes da Silva[3], Matheus Ribeiro Prado[3], Murilo Alves Beppler[2], and Anderson Rodrigues dos Santos[2,*]

[1]Biotechnology Institute, Federal University of Uberlândia, Rua Acre, 104, Uberlândia, 38405-319, Minas Gerais, Brazil
[2]Faculty of Computing, Federal University of Uberlândia, Av. João Naves de Ávila, 2121, Uberlândia, 38400-902, Minas Gerais, Brazil
[3]Information Management Department, Federal University of Uberlândia, Av. João Naves de Ávila, 2121, Uberlândia, 38400-902, Minas Gerais, Brazil
[*]Corresponding author
[†]These authors contributed equally to this work.

Corresponding author: santosardr@ufu.br

Abstract

While renowned for mitigating methane emissions via anaerobic oxidation (AOM), the full ecological strategy of ANME-2a archaea still requires further exploration. This study looks beyond methane oxidation to map the protein landscape of ANME-2a, revealing an integrated system for metabolic autonomy and environmental resilience in this specific isolate. Using a feature-based protein network derived from 230 Methanosarcinales genomes, we uncovered a sophisticated modular architecture. Key findings demonstrate that the AOM machinery is not isolated but functionally coupled with distinct modules dedicated to auxiliary functions. Our analysis not only confirms that this ANME-2a isolate possesses the complete genomic toolkit for autonomous diazotrophy but also reveals the molecular blueprint for its integration with AOM. We show how key machinery has been specialized to support the organism's core energy metabolism, with its core nitrogenase components co-clustered within the same functional module as AOM electron transfer proteins. Furthermore, we identified a specialized module dedicated to "membrane fortification" through the significant enrichment of pathways for archaeal lipid biosynthesis. This modular blueprint, which integrates core energy metabolism with nitrogen fixation and structural lipid production, provides a model for how diazotrophic ANME-2a lineages may thrive as robust, self-sufficient organisms adapted to dynamic, resource-limited ecosystems.

Keywords: ANME-2a, Anaerobic methane oxidation (AOM), Protein network, Diazotrophy, Archaeal lipids, Metabolic modularity, Niche adaptation

Appendix A Degree Distribution Analysis of the ANME-2a PPI Network

We investigated the topological properties of the constructed archaeal protein interaction network by analysing its node degree distribution. We computed the complementary cumulative distribution function (CCDF), $P(K \geq k)$, which represents the probability that a node has a degree K greater than or equal to k from the observed degrees. This CCDF was visualised on logarithmic axes to assess the distribution's tail behaviour (Figure 1).

To quantitatively characterise the distribution's tail, we fitted two candidate discrete models to the data using Maximum Likelihood Estimation (MLE), as implemented in the `poweRlaw` R package (Gillespie, 2015): (i) a pure power-law (PL) distribution, characterised by $P(k) \propto k^{-\gamma}$, and (ii) a discrete lognormal (LN) distribution. The optimal lower degree threshold (`xmin`) for initiating the power-law fit was estimated using the methods described by (Clauset et al., 2009), yielding `xmin` $= 7$.

Fitting the power-law model to the distribution's tail ($k \geq 7$) via Maximum Likelihood Estimation yielded an exponent $\gamma \approx 1.65$ (Figure 1, red solid line). Fitting the discrete lognormal model to the same data range ($k \geq 7$) resulted in estimated parameters $\mu \approx 2.53$ and $\sigma \approx 1.58$ (Figure 1, green dashed line).

We performed a formal statistical comparison using Vuong's test to determine which model better describes the observed tail data. These results showed a statistically significant preference for the power-law model over the lognormal model in describing the data for $k \geq 7$ (Vuong's Test Statistic ≈ -10.8, $p \approx 3.0 \times 10^{-27}$). This finding indicates that, within the analysed tail region, the degree distribution is most consistent with a power law, supporting the characteri

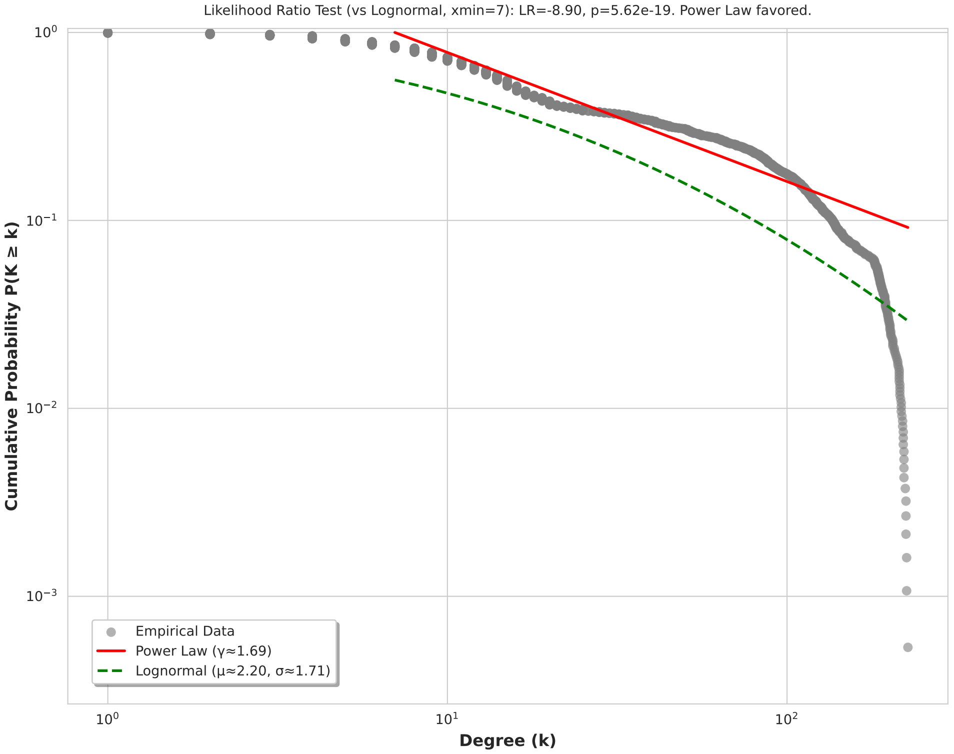

Figure 1: Log-log plot of the complementary cumulative degree distribution (CCDF) for the archaeal protein interaction network. Grey points show the empirical probability $P(K \geq k)$ that a node has a degree K greater than or equal to k. Lines represent model fits estimated via Maximum Likelihood Estimation for the tail of the distribution beginning at $k \geq 7$. The red solid line corresponds to the pure power-law fit (estimated exponent $\gamma \approx 1.65$). The green dashed line corresponds to the discrete lognormal fit.

Appendix B Detailed Manual Curation of Selected Protein Interactions

We manually curated a selection of proteins within the AOM network and their predicted interactions to gain deeper functional insights beyond those provided by automated annotations. This process highlighted several proteins and potential interactions of significant biological relevance to AOM and its associated processes:

Potential Mediators of Electron Transfer: Our manual analysis corroborated the presence of numerous auxiliary redox proteins within the AOM net, featuring domains like Fe-S clusters (e.g., MRG76146.1, MRG76886.1, MRG76970.1), FMN-binding sites (e.g., MRG76149.1), and ferredoxin/rubredoxin motifs (e.g., MRG76285.1). These proteins interact with core AOM components (Rnf, MHC, Hdr), suggesting a complex network that supports or supplements the primary electron transfer pathways. Notably, our curati

Bioenergetic Support and Regulation: We uncovered interactions suggesting links between AOM bioenergetics and cellular energy status. For instance, we predicted MRG77858.1, a CDC48 family AAA ATPase found in ETRC, to interact with the membrane-bound HdrDE complex (containing canonical AOM protein MRG76549.1 in ETRC). This interaction points towards potential ATP-dependent modulation of HdrDE activity, possibly related to its assembly, function in ion translocation, or regulation (Meyerdierks et al., 2010)

Connection to Cofactor Biosynthesis and Stress Response: Our manual curation reinforced connections to essential metabolic processes supporting AOM. We found MRG76964.1 (a bridging protein in CC1M), annotated as a dihydroneopterin aldolase involved in the biosynthesis of the vital C1 carrier H4MPT, interacting with Hdr subunits (canonical, in ETRC/CC1M), suggesting proximity or coordination between cofactor supply and electron transfer components. Additionally, MRG76144.1 (in MFCB), possessing a

Hypothetical Proteins with Intriguing Links: Our curation process also flagged several hypothetical proteins or those with domains of unknown function (DUFs) that exhibited strong predicted interactions with core AOM machinery or proteins in key pathways, highlighting them as priority targets for future characterization. Examples include MRG76155.1 (in ETRC) with a putative Zn/Fe chelating domain interacting with MHC (canonical, in APCR) and MRG76545.1 (in CC1M) containing a DUF126/Aconitase X d

These curated observations provide specific examples and functional hypotheses that enrich the understanding derived from pathway analysis, pointing towards specific mechanisms for electron transfer, energy coupling, regulation, and adaptation within the AOM net of ANME-2a.

Appendix C Detailed Bridging Centrality Analysis and Key Integrating Proteins

Network topology analysis provides a quantitative means to identify influential nodes within protein-protein interaction networks. Various centrality metrics highlight different aspects of a node's importance. In this study, we specifically employed bridging centrality calculated on the feature-based AOM net. This metric quantifies the extent to which a node lies on the shortest paths connecting nodes belonging to different, densely connected communities (modules). Therefore, nodes with high bridging centrality scores are likely to serve as key integrators that facilitate inter-module communication and coordination within the network.

MRG76891.1 (NAD(P)H-quinone oxidoreductase subunit)

As a canonical component of the AOM pathway (part of Fpo/Fqo in module CC1M), the membrane-bound NAD(P)H-quinone oxidoreductase subunit MRG76891.1 is integral to energy conservation via electron transport. Its interactions bridge this core membrane bioenergetic machinery, including connections to other putative subunits of the same complex located in the membrane (NuoK, in CC1M) or predicted as surface-exposed (NuoH, in CC1M) to a diverse suite of cytoplasmic functions predominantly associated with biosynthesis and regulation (components across multiple modules). This positioning makes MRG76891.1 a

critical nexus for coordinating energy generation with cellular biosynthetic and regulatory demands.

MRG77314.1 (Type II methionyl aminopeptidase)

The cytoplasmic Type II methionyl aminopeptidase (MRG77314.1) within module CC1M is a central hub for post-translational protein maturation. Its interactions position it as a key integrator, directly linking its enzymatic function to the cytoplasmic protein synthesis machinery (e.g., tRNA ligases in CC1M) and core AOM enzymes (such as Ftr and Mtr subunits, also cytoplasmic within CC1M). Furthermore, it bridges these core processes to broader biosynthetic pathways (components in CC1M and MFCB), reflecting its crucial role in maintaining the cellular proteome that supports AOM function.

MRG76974.1 (AAA family ATPase)

Located in module CC1M, the cytoplasmic AAA family ATPase (MRG76974.1) appears to provide a direct energetic and regulatory link between genome maintenance and core AOM bioenergetics. It interacts with components of the DNA repair and metabolism machinery (such as cytoplasmic exonuclease SbcD and DNA polymerase II subunit within CC1M) and, notably, with the Heterodisulfide Reductase (Hdr, MRG76973.1, a canonical AOM protein in CC1M), a key AOM enzyme also predicted here as potentially surface-exposed. This unique positioning suggests that MRG76974.1 coordinates cellular genome maintenance activities with the energy status reflected by AOM pathway activity, potentially serving as a regulatory checkpoint that links DNA repair capacity to metabolic flux through the AOM pathway.

MRG76964.1 (Hypothetical protein)

The cytoplasmic hypothetical protein MRG76964.1 in module CC1M functions as a critical inter-module bridge, connecting distinct facets of C1 and redox metabolism, thereby supporting the functional partitioning we observed between modules. It links the core AOM bioenergetic machinery centred in CC1M, including interactions with cytoplasmic and potentially surface-exposed subunits of the Heterodisulfide Reductase (Hdr) complex (canonical AOM proteins in CC1M and ETRC), to enzymes associated with one-carbon metabolism and cofactor synthesis predominantly found in MFCB. This bridging function is particularly significant because it suggests coordinated regulation between the core energy-generating AOM pathway and the synthesis of essential cofactors required for its operation.

MRG76793.1 (TIGR00297 family protein)

Predicted as surface-exposed (PSE) and residing within module CC1M, the TIGR00297 family protein MRG76793.1 is a critical link between the cell's external interface and internal machinery, including a core AOM component. It forms connections with a cluster of other PSE proteins likely involved in transport or sensing at the cell surface (such as a tungstate ABC transporter substrate-binding protein and an FtsX-like permease), bridging this external complex to the diverse cytoplasmic hub of biosynthesis and regulation within CC1M. This dual connectivity suggests that MRG76793.1 may serve as a sensor or transducer that coordinates the cell's metabolic state with environmental conditions, potentially modulating AOM activity based on substrate or cofactor availability.

MRG77469.1 (Ligand-binding protein SH3)

Located at the cell membrane and assigned to module CC1M, the SH3 domain-containing protein MRG77469.1 acts as a potential scaffolding or signalling adapter, bridging membrane-associated events with the cytoplasmic network. Its SH3 domain likely mediates protein-protein interactions, connecting it to the extensive cytoplasmic hub of core metabolic (Fmd, a canonical AOM protein in CC1M), DNA maintenance (RFC, repair enzymes, in CC1M), redox (thioredoxin reductase, in CC1M), and signalling (NTP transferase, in CC1M) functions within CC1M. This multifaceted connectivity positions MRG77469.1 as a key coordi-

nator that integrates membrane-associated signal transduction with the diverse cytoplasmic processes essential for AOM function and cellular homeostasis.

Appendix D Comparative Genomic Analysis of Key Functional Modules

To contextualise the findings from the reference genome (*Candidatus Methanocomedens sp.*, a comparative analysis was performed using BLASTp to search for key proteins from identified functional modules in four other publicly available ANME-2a genomes. The query proteins were selected as essential representatives of each module, with lengths ranging from 248 to 655 amino acids. The results, summarised in Table 1, were interpreted based on the percentage sequence identity and alignment coverage of the best BLASTp hit.

Table 1: Quantitative summary of the comparative genomic analysis across ANME-2a genomes. Each cell shows the per cent sequence identity of the best BLASTp hit. Hits were considered high-confidence if they covered >90% of the query sequence. Full-length query sequences are detailed in the manuscript methods.

Functional Module	Query Protein	GCA_013374385 (1237 proteins)	GCA_013374455 (2467 proteins)	GCA_013374465 (2008 proteins)	GCA_013572335 (1462 proteins)
AOM Central (Control)	McrA (575 aa)	Not Found	98.8%	Not Found	97.2%
	McrB (434 aa)	Not Found	99.5%	Not Found	95.4%
	McrG (248 aa)	Not Found	100.0%	Not Found	99.6%
Nitrogen Fixation	NifB (296 aa)	90.9%	25.1%[a]	100.0%[b]	91.2%
	NifH (274 aa)	Not Found	72.2%	Not Found	Not Found
	NifD (526 aa)	Not Found	43.9%	Not Found	Not Found
	NifK (456 aa)	Not Found	42.0%	Not Found	Not Found
	NifE (475 aa)	Not Found	42.7%	Not Found	Not Found
	NifN (457 aa)	Not Found	38.2%	Not Found	Not Found
Membrane Fortification	HMG-CoA synthase (346 aa)	83.8%	99.7%	Not Found	93.9%
	HMG-CoA reductase (412 aa)	74.5%	100.0%	Not Found	93.7%
	Mevalonate kinase (298 aa)	76.5%	98.3%	98.7%[c]	89.3%
	GG-P synthase (404 aa)	67.4%	97.8%	Not Found	90.1%
Secondary Metabolism	Radical SAM (393 aa)	86.0%	99.5%	99.0%	93.1%
	Acetate-CoA ligase (655 aa)	79.0%	98.8%	98.0%	92.8%

[a]Weak hit: low identity and partial coverage (~75%). Interpreted as a non-functional paralogue.
[b]High-confidence hit, but alignment covers only 78% of the query length.
[c]High-confidence hit, but alignment covers only 75% of the query length.

Interpretation of Comparative Results

The quantitative analysis reveals distinct conservation patterns, where high-confidence orthologues were identified based on high sequence identity (>65%) and alignment coverage exceeding 90% of the query protein length.

- Nitrogen Fixation: The machinery for diazotrophy appears to be a variable or 'accessory' trait within the ANME-2a clade. The most complete genome, `GCA_013374455`, possesses clear orthologues for the entire *nifHDKEN* structural operon (38-72% identity, all with >95% query coverage). However, the best hit for the essential *nifB* gene in this genome was a weak, partial match (25.1% identity over ~75% of the query), which we interpret as a distant paralog rather than a functional ortholog.
- Membrane Fortification: The core enzymes for archaeal lipid biosynthesis are highly conserved across nearly all analysed genomes. The identified orthologues show very high sequence identity (74-100%) and near-complete alignment coverage, suggesting this module is a fundamental adaptive trait. The notable exception is genome `GCA_013374465`, which, despite its relatively high number of predicted proteins, appears to be missing most of this pathway. This finding could indicate genome incompleteness or suggest that alternative membrane composition strategies exist within the clade.
- Secondary Metabolism: The representative proteins from the secondary metabolite cluster enriched in our network were found in all four comparative genomes with very high identity (79-99%) and full alignment coverage. This high degree of conservation, even in the most fragmented genomes, suggests that the capacity to produce these metabolites may be a core feature of the ANME-2a clade, potentially playing an important role in ecological interactions.

Appendix E Functional Annotation of Proteins in the MFCB Module with Assigned KOs

Table 2: Detailed functional annotation of proteins within the Membrane Fortification and Central Biosynthesis (MFCB) module that were assigned KEGG Orthology (KO) identifiers. Proteins are grouped by their primary contribution to the module's name. Superscripts indicate the protein's primary role: ^{MF} for Membrane Fortification; ^{CB} for Central Biosynthesis.

Protein ID	Gene / Abbreviated Product	KO ID	KO Description (Representative KEGG Pathway)
Evidence for Membrane Fortification (MF)			
MRG76194.1^{MF}	methyltransferase domain-containing...	K03183	Ubiquinone and other terpenoid-quinone biosynthesis
Evidence for Central Metabolism (CM)			
MRG76696.1^{CM}	citrate/2-methylcitrate synthase	K01647	Citrate cycle (TCA cycle)
MRG77002.1^{CM}	sdhB	K00240	Citrate cycle (TCA cycle)
MRG77101.1^{CM}	FAD-dependent oxidoreductase	K00239	Citrate cycle (TCA cycle)
MRG77049.1^{CM}	oadA	K01960	Carbon metabolism
MRG77050.1^{CM}	acetyl-CoA carboxylase...	K01959	Carbon metabolism
MRG77102.1^{CM}	aspartate ammonia-lyase	K01679	Carbon metabolism
MRG77777.1^{CM}	aldehyde ferredoxin oxidoreductase	K03738	Carbon metabolism
MRG77941.1^{CM}	2-oxoacid ferredoxin oxidoreductase	K00175	Carbon metabolism
Biosynthesis of Building Blocks (Amino Acids, Cofactors, etc.)			
MRG76179.1^{CM}	aminotransferase class I/II-fold...	K00812	Alanine, aspartate and glutamate metabolism

Continued on next page

Table 2: Functional annotation of proteins in the MFCB module (Continued)

Protein ID	Gene / Abbreviated Product	KO ID	KO Description (Representative KEGG Pathway)
MRG76799.1[CM]	folP	K00796	Folate biosynthesis
MRG76880.1[CM]	2-dehydropantoate 2-reductase	K00077	Pantothenate and CoA biosynthesis
MRG76675.1[CM]	glutamate–tRNA ligase	K01885	Aminoacyl-tRNA biosynthesis
MRG76321.1[CM]	ROK family protein	K00845	Amino sugar and nucleotide sugar metabolism
		K00847	Fructose and mannose metabolism
		K00884	Amino sugar and nucleotide sugar metabolism
MRG77241.1[CM]	bifunctional precorrin-2...	K02304	Porphyrin metabolism
MRG77242.1[CM]	glutamyl-tRNA reductase	K02492	Porphyrin metabolism
MRG77289.1[CM]	ltrA	K00986	Biosynthesis of secondary metabolites
Other Supporting Cellular Processes			
MRG76219.1	P-loop NTPase	K03609	General function prediction only
MRG76310.1	rsgA	K06949	Thiamine metabolism
MRG76386.1	peptidylprolyl isomerase	K01802	General function prediction only
		K03775	General function prediction only
MRG76485.1	type II secretion system F...	K07333	General function prediction only
MRG76523.1	nrdD	K21636	Purine metabolism
MRG76666.1	AAA family ATPase	K07133	General function prediction only
MRG76748.1	DNA-3-methyladenine glycosylase...	K03660	Base excision repair
MRG76846.1	ATPase	K06865	General function prediction only
MRG76941.1	chemotaxis protein CheB	K03412	Bacterial chemotaxis
MRG77029.1	DEAD/DEAH box helicase	K03725	General function prediction only
MRG77104.1	helix-turn-helix domain-containing...	K07494	General function prediction only
		K07499	General function prediction only
MRG77145.1	tRNA (N6)-L-threonylcarbamoyl...	K15865	General function prediction only
MRG77232.1	ATP-binding cassette...	K02031	Quorum sensing
		K02032	Quorum sensing
MRG77371.1	TSUP family transporter	K07090	General function prediction only
MRG77389.1	pan	K03420	Proteasome
MRG77424.1	hypothetical protein	K07331	General function prediction only
MRG77562.1	secretion system protein	K07333	General function prediction only
MRG77597.1	uvrC	K03703	Nucleotide excision repair
MRG77659.1	TIGR00295 family protein	K06950	General function prediction only
MRG77726.1	AAA family ATPase	K06921	General function prediction only
MRG77746.1	DUF541 domain-containing...	K09807	General function prediction only
MRG77808.1	ABC transporter substrate-binding...	K02016	General function prediction only
MRG77818.1	Na(+)/H(+) antiporter...	K05568	General function prediction only
MRG77869.1	DUF460 domain-containing...	K09150	General function prediction only
MRG77970.1	ATP-binding cassette...	K16786	ABC transporters
		K16787	ABC transporters
MRG77971.1	ATP-binding cassette...	K16787	ABC transporters

Continued on next page

Table 2: Functional annotation of proteins in the MFCB module (Continued)

Protein ID	Gene / Abbreviated Product	KO ID	KO Description (Representative KEGG Pathway)
MRG78014.1	cytochrome C biogenesis...	K06196	General function prediction only

References

Aaron Clauset, Cosma Rohilla Shalizi, and M. E. J. Newman. Power-Law Distributions in Empirical Data. *SIAM Review*, 51(4):661–703, 2009. DOI: 10.1137/070710111. URL https://doi.org/10.1137/070710111.

Colin S Gillespie. Fitting Heavy Tailed Distributions: The poweRlaw Package. *Journal of Statistical Software*, 64(2):1–16, 2015. URL https://cran.r-project.org/web/packages/poweRlaw/vignettes/d_jss_paper.pdf.

Anke Meyerdierks, Michael Kube, Ivaylo Kostadinov, Hanno Teeling, Frank Oliver Glockner, Richard Reinhardt, and Rudolf Amann. Metagenome and mRNA expression analyses of anaerobic methanotrophic archaea of the ANME-1 group. *Environmental Microbiology*, 12(2):422–439, February 2010. ISSN 1462-2920. DOI: 10.1111/j.1462-2920.2009.02083.x.
