# Supplementary figures and images for: Beyond Methane Oxidation: The Protein Landscape of ANME‐2a Reveals an Integrated System for Diazotrophy and Membrane Fortification
Source: Environ Microbiol Rep. 2025 Nov 17;17(6):e70233. doi: 10.1111/1758-2229.70233 (PMC12622379; doi:10.1111/1758-2229.70233)

Likelihood Ratio Test (vs Lognormal, xmin=7): LR=-8.90, p=5.62e-19. Power Law favored.

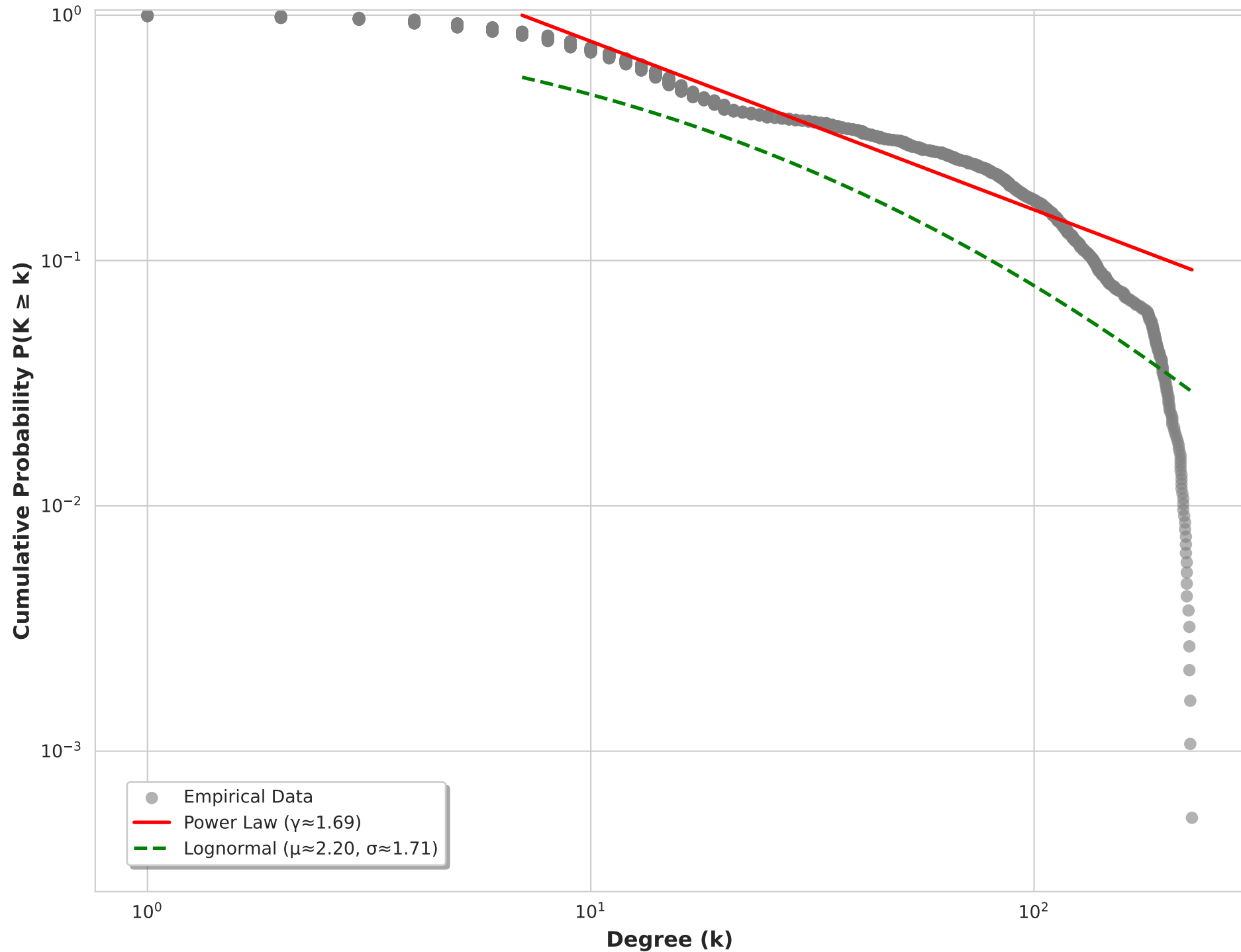

Supplement: Supplementary file 1 — Data S1: emi470233‐sup‐0001‐supinfo.zip. [file EMI4-17-e70233-s001.zip › ccdf_pl_vs_lognormal.pdf]
